# Severe atopic dermatitis with cutis laxa caused by a variant in the *ELN* gene

**DOI:** 10.1016/j.jdcr.2024.01.029

**Published:** 2024-02-13

**Authors:** Tatsuya Katsumi, Ryota Hayashi, Shingo Takei, Rei Yokoyama, Osamu Ansai, Satoru Shinkuma, Riichiro Abe

**Affiliations:** a Division of Dermatology, Niigata University Graduate School of Medical and Dental Sciences, Niigata, Japan; b Department of Dermatology, Nara Medical University, Kashihara, Japan

**Keywords:** atopic dermatitis, cutis laxa, dupilumab, *ELN* gene

## Introduction

Cutis laxa (CL) is a group of rare connective tissue disorders characterized by loose and hypoelastic skin.^1^ There are both inherited and acquired forms of CL and the forms of autosomal dominant and autosomal recessive is exist in the inherited pattern of CL.^1^ The *ELN* gene is one of the causative gene for autosomal dominant form of CL (ADCL, OMIM: 123700).^1^ The patients with ADCL have generalized loose skinfolds.^1^ Although wrinkles with lichenification are also observed in atopic dermatitis (AD). We herein report a case of severe AD with CL caused by a variant in the *ELN* gene.

## Case report

A 27-year-old Japanese woman was originally suspected with CL and she has had facelift surgeries 3 times performed by plastic surgeons when she was child. She had presented with severe AD since childhood and had been treated with corticosteroid ointment. However, her skin manifestations did not improve. She was referred to our hospital for additional treatment for AD.

**Fig 1:**
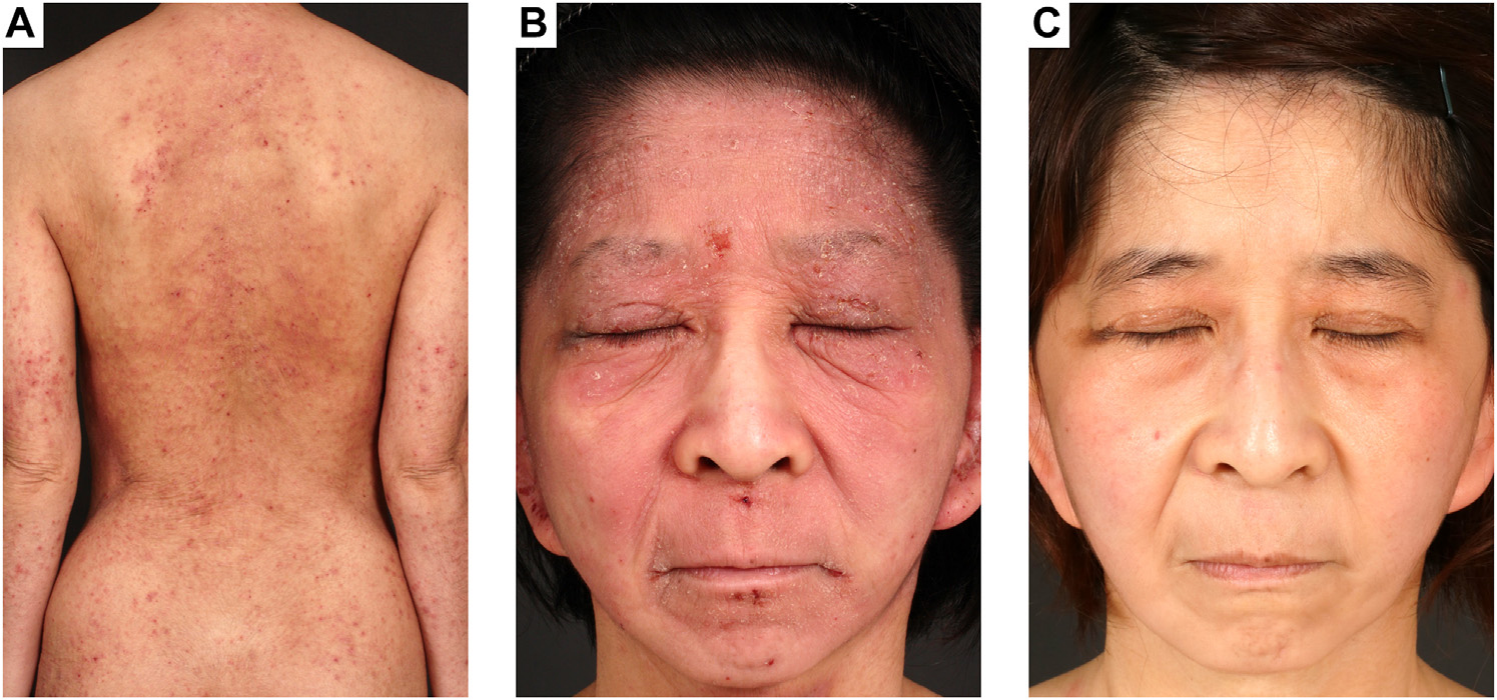
**A,B,** Physical examination before dupilumab treatment. **A**, There was scaly erythema with lichenification on her back. **B**, Loose skin with scaly erythema was shown especially on her cheek. **C**, Physical examination after dupilumab treatment. Her loose skin was dramatically improved by dupilumab treatment.

**Fig 2:**
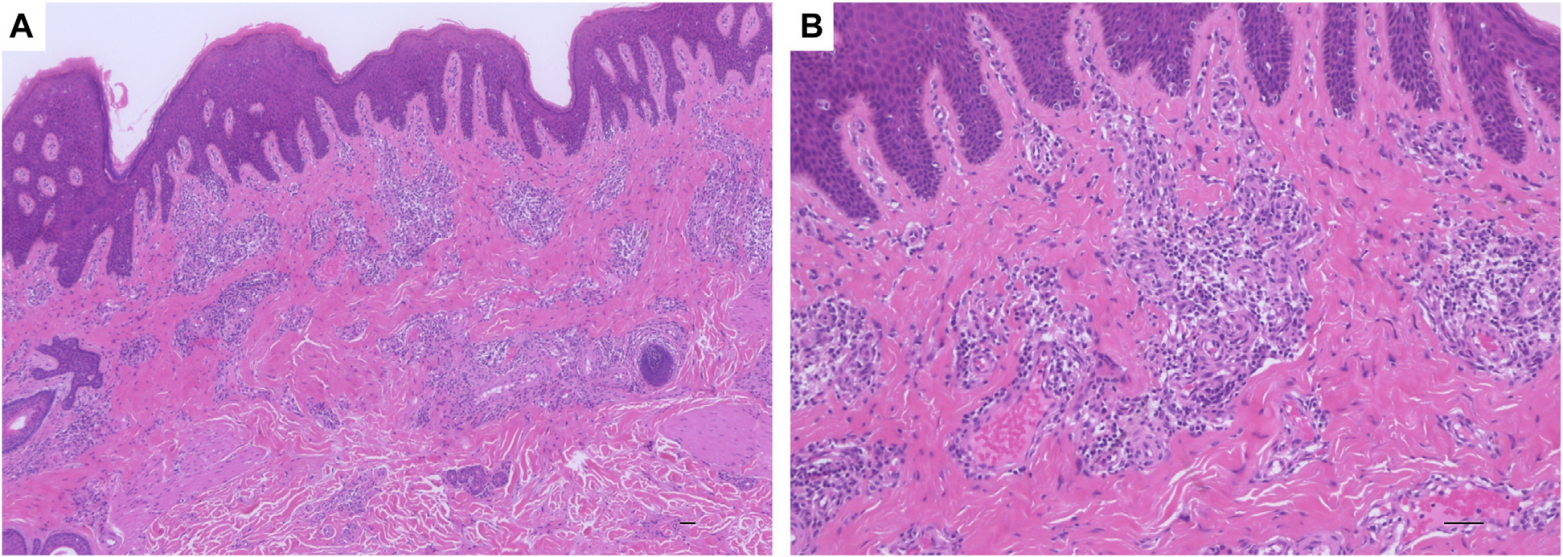
**A,B,** In pathologic findings, there was acanthosis and inflammatory cells around the appendages in the papillary dermis. Scale bar: 50 μm.

**Fig 3:**
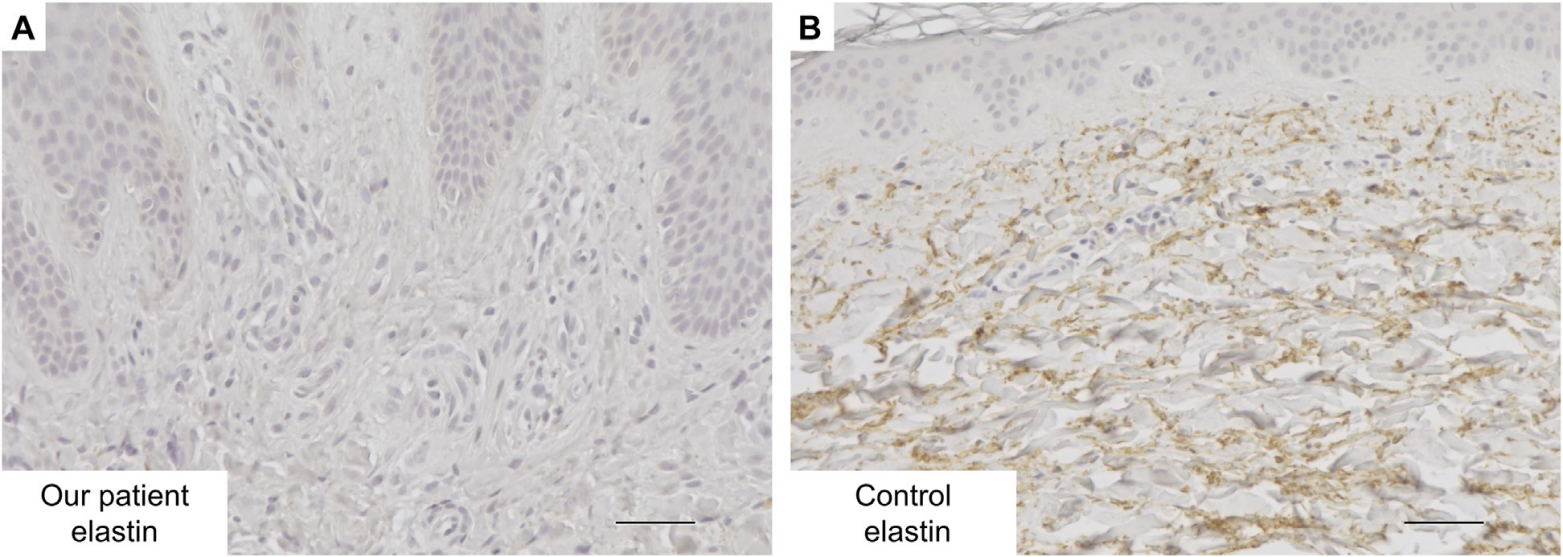
**A,B,** Immunohistochemical examination of elastin showed decrease of dermal elastic fibers in this patient (**A**) compared with normal control (**B**). Scale bar: 50 μm. Those stained with DAB (3,3′diaminobenzidine) are positive for elastin.

On physical examination, scaly erythema with lichenification was on her entire body (Fig 1, *A*, *B*). Moreover, loose skin was shown especially on her cheek and neck, and she was an aged appearance (Fig 1, *A*, *B*). There was no history of cardiovascular diseases. Her laboratory data when she was 25-year-old showed white blood cell 9050/μL (eosinophil 8.7%), IgE 33,700 IU/mL, and thymus and activation-regulated chemokine 4086 pg/mL. In pathologic findings, there was acanthosis and inflammatory cells around the appendages in the papillary dermis (Fig 2, *A*, *B*). Immunohistochemical examination of the facial skin showed a decrease of elastic fibers in the dermis (Fig 3, *A*, *B*). To diagnose ADCL, we performed whole exome sequencing and identify a novel deletion variant c.2345delG (p.Gly782Alafs∗30) in exon 34 of the *ELN* gene (NM_001278939.2). We detected a recurrent nonsense variant c.12064A>T(p.Lys4022∗) of the *FLG* gene (NM_002016.2). Based on clinical findings and mutation analysis, we diagnosed her with ADCL and AD. She was treated with dupilumab for symptoms of AD and her symptoms gradually improved. Unexpectedly, her wrinkles also dramatically improved (Fig 1, *C*). After 6 months of treatment, the Eczema Area and Severity Index score significantly decreased (from 30.8 to 3.15). At this stage, after 5 years of use of dupilumab, there have been no adverse events and AD is well controlled.

## Discussion

The human elastin gene contains 34 exons in its longest transcript and ADCL is predominantly caused by frameshift mutations in exons 30 to 34.^1^ In general, mutant mRNA in patients with ADCL is stable and in some, protein containing the frameshifted alleles has been detected in the matrix using an antibody to the frameshifted product, suggesting a dominant negative mechanism of disease.^1^ Approximately 20 *ELN* mutations have been reported in ADCL, the relationship between ADCL and AD has not been clarified,^2^^,^^3^ and we considered AD incidentally occurring in our patient. However, we think that the degree of loose skin and edema is more severe in patients with AD and ADCL than in with only AD. Immunohistochemical examination of elastin showed less expression of dermal elastic fibers in our case than in previous reports.^2^ Previous reports have suggested that different levels of mutant mRNA correlated with the severity of the clinical presentation.^4^

ADCL caused by *ELN* variants was initially thought to affect only the skin. However, a recent study revealed that 30% to 50% of patients with ADCL presented with aortic root dilation and other pulmonary diseases.^1^ Recently, a case of critical stenosis in a 21-year-old woman with an intronic mutation in the *ELN* gene was reported.^5^ The patient was originally diagnosed with ADCL however the causative gene was never identified. Therefore, patients with *ELN* gene mutations should also be followed for cardiac disease.

As for wrinkles, the Dennie-Morgan fold is generally known as one of a symptom of AD; however; the presence of CL further increases wrinkles. In the patient, severe loose skin especially observed on her face before the treatment for dupilumab. Whereas in areas other than the wrinkle-prone face, loose skin was not improved by dupilumab. Therefore, we considered dupilumab did not directly improve loose skin and the administration of dupilumab to patients with ADCL and AD might be useful in improving facial severe symptoms.

## Conflicts of interest

None disclosed.
